# Smaller-the-better-type six sigma product index

**DOI:** 10.1038/s41598-023-44721-3

**Published:** 2023-10-19

**Authors:** Kuen-Suan Chen, Tsung-Hua Hsieh, Chun-Min Yu, Kai-Chao Yao

**Affiliations:** 1 Department of Industrial Engineering and Management, National Chin-Yi University of Technology, Taichung, 411030 Taiwan, ROC; 2 Department of Business Administration, Chaoyang University of Technology, Taichung, 413310 Taiwan, ROC; 3 Department of Business Administration, Asia University, Taichung, 413305 Taiwan, ROC; 4 Department of Industrial Education and Technology, National Changhua University of Education, Changhua, 50074 Taiwan, ROC; 5 Department of Leisure Service Management, Chaoyang University of Technology, Taichung, 413310 Taiwan, ROC

**Keywords:** Engineering, Mathematics and computing

## Abstract

Based on some studies, there are many important parts of tool machines, all of which have some essential smaller-the-better-type quality characteristics. The six sigma quality index of the smaller-the-better type offers accurate measurement of the process yield and the six sigma quality level. In this paper, we first proposed a six sigma product index by integrating all evaluation indicators for products that contain several quality characteristics of the smaller-the-better type. Next, we derived the confidence interval of this six sigma product index and developed an evaluation model for product quality. When a product passes the evaluation of this model, not only can it be guaranteed that the product reaches the required quality level, but also a high rate of product yield can be ensured. In addition, we also created a product improvement testing model, which can avoid missing opportunities for improvement in the process to ensure improvement effects. This complete evaluation and improvement model is applicable to the entire machine tool industry chain. It can not only increase the product value of the machine tool industry chain but also decrease environmental pollution caused by rework or scrap, which is beneficial to companies to enhance their image of fulfilling social responsibilities. Apart from the above advantages, the model formed in this paper is based on confidence intervals, thereby reducing the chance of misjudgment resulting from sampling error.

## Introduction

The output value and export volume of the industries related to Taiwan’s machine tools are among the best in the world, and their products are mainly sold to countries of emerging markets, such as Southeast Asia and Eastern Europe. According to many studies, about 70% of machine tools and their component processing factories are located in the central region, and central Taiwan has formed a large industrial cluster of machine tools^1,2^. The machine tool industry and its suppliers in this industrial cluster as well as machine tool purchasers scattered all over the world form a machine tool industry chain^3,4^. Numerous studies have pointed out that a product usually has multiple key quality characteristics. For products with multiple quality characteristics, the process capability of all quality characteristics must reach the required level, so that the final product quality of the component can be ensured^5,6^. For example, when the concentricity of the axis is not good, it will cause the assembled machine tool product to shake or vibrate during operation, which not only affects the precision and accuracy of the machine tool processing but also shortens the service life of the machine tool.

According to some studies, many important parts of tool machines have some important quality characteristics of the smaller-the-better (STB) type^7^. As noted by Luo et al.^8^ and Yu et al.^9^, common STB-type quality characteristics include roundness, concentricity, perpendicularity, and surface roughness of gears, bearings, shafts, etc. In addition, a number of studies have indicated that enhancing product quality can not only raise product value but also reduce rework and scrap, thereby decreasing maintenance rates^10–12^. Clearly, reduction of rework and scrap can lower carbon emissions and environmental pollution^13,14^. Thus, this paper proposes a complete evaluation and improvement model for a number of component products containing the STB quality characteristics, which will help improve the product quality of machine tools as well as help enhance the quality of component products processed by machine tools at the same time. Not only can this model elevate product value in the industry chain, but it also can diminish environmental pollution caused by rework or scrap, which is beneficial for enterprises to enhance the image of fulfilling social responsibility.

In order not to lose generality, this paper assumes that the components processed by machine tools have STB-type quality characteristics. For these STB-type quality characteristics, the STB-type six sigma quality index proposed by Chang et al.^15^ offers accurate measurement of the process yield and the six sigma quality level. Consequently, this paper uses this STB six sigma quality index as the evaluation index for the STB-type quality characteristics. Then, a six sigma product index that has a one-to-one mathematical relationship with the product yield is proposed by means of integrating all the evaluation indexes of the STB-type quality characteristics. First, this paper derives the 100($1 - \alpha$)% confidence intervals of the six sigma quality index for all quality characteristics based on in-control data. Next, the 100($1 - \alpha$)% confidence intervals of the six sigma product index are derived based on all confidence intervals of the six sigma quality index. Afterward this paper uses the confidence intervals derived above to evaluate whether the component products meet the required quality level. Based on the confidence intervals, the risks of misjudgment caused by sampling error can be lowered. When the evaluation result does not meet the requirements of the quality level, the process must be improved. Subsequently, the confidence intervals of the six sigma product index before and after improvement are employed to examine the quality levels before and after improvement and see whether there is any significant difference, in order to verify the improvement effectiveness.

Based on the above, the proposed evaluation and improvement model for products with multiple smaller-the-better-type quality characteristics has the following advantages and contributions: (1) The suggested six sigma product index can simultaneously reflect the six sigma quality level and process yield. (2) Based on the confidence intervals, the risk of misjudgment caused by sampling error can be diminished. (3) It is applicable to the entire machine tool industry chain. It can not only raise the product value of the machine tool industry chain but also lower the environmental pollution incurred by rework or scrap. Moreover, it is conducive to improving the image of enterprises in fulfilling their social responsibilities.

The other sections in this paper are organized as follows. Section “Six sigma quality index and six sigma product index” introduces the six sigma quality index and the six sigma product index. In Sect. “Confidence intervals”, we derive the 100(1 $$-$$$$\alpha$$)% confidence intervals of the six sigma quality index; based on the confidence intervals of the six sigma quality index, the confidence intervals of the six sigma product index are retrieved from the mathematical programming method. Then, we propose a quality evaluation and improvement testing model for component products in Sect. “Quality Evaluation and Improvement Testing Model of Component Products”. An illustrative example is presented in Sect. “An illustrative example” to demonstrate the applicability of the proposed approach. Finally, Sect. “Conclusions and Future Scope of Research” provides the conclusions.

## Six sigma quality index and six sigma product index

As mentioned above, without losing generality, this paper assumes that the component product processed by machine tools has STB-type quality characteristics. Let random variable $X_{h}$ be the normal process distribution of the STB-type quality characteristic *h*, denote by $X_{h} \sim N\left( {\mu_{h} ,\sigma_{h}^{2} } \right)$, *h* $=$ 1, 2, …, s. According to Chen et al.^17^, the target value of the STB quality characteristics is zero ($T_{h}$ $=$ 0). Thus, process mean $\mu_{h}$ can only be larger than target value $T_{h}$, which means it is impossible to be smaller than target value $T_{h}$. In practice, based on cost consideration and limitations of the processing technology, the measured value of the product is usually far from target value $T_{h}$, that is, it is much larger than 0 and usually very close to the upper limit of the specification, so process standard deviation $\sigma_{h}$ is relatively small^15–17^. According to Grau^18^, the target value may not be always zero. Thus, we can let random variable be $Y_{h} = {{\left( {X_{h} - T_{h} } \right)} \mathord{\left/ {\vphantom {{\left( {X_{h} - T_{h} } \right)} {d_{h} }}} \right. \kern-0pt} {d_{h} }}$, where $d_{h} = USL_{h} - T_{h}$. Even so, for STB-type quality characteristics, the ideal target value is zero ($T_{h} = 0$). In this case,$Y_{h} = {{X_{h} } \mathord{\left/ {\vphantom {{X_{h} } {USL_{h} }}} \right. \kern-0pt} {USL_{h} }}$, where $Y_{h}$ is distributed as a normal distribution with mean $\delta_{h}$ and standard deviation $\gamma_{h}$, and $USL_{h}$ is the upper specification limit of quality characteristic *h*, denote by $Y_{h} \sim N\left( {\delta_{h} ,\gamma_{h}^{2} } \right)$, *h* $=$ 1, 2, …, *s*. Based on Chang et al.^15^, the STB-type six sigma quality index of quality characteristic *h* can be shown as follows:

1 $$Q_{S - T - B,h} \, = \,\frac{{{1} - \delta_{h} }}{{\gamma_{h} }}.$$

In addition, the probability of $\left( {X \le USL} \right)$ is equal to the probability of $\left( {Y \le 1} \right)$. Then, process yield $p_{h}$ is defined as follows:

2 $$p_{h} \, = \,p\left\{ {Z \le \frac{{1 - \delta_{h} }}{{\gamma_{h} }}} \right\} = F_{Z} \left( {Q_{S - T - B,h} } \right),$$

where $Z = {{\left( {Y - \delta_{h} } \right)} \mathord{\left/ {\vphantom {{\left( {Y - \delta_{h} } \right)} {\gamma_{h} }}} \right. \kern-0pt} {\gamma_{h} }}$. Obviously, the process yield has a one-to-one mathematical relationship with the six sigma quality index $Q_{S - T - B,h}$. Under the assumption of normality,

3 $$p_{h} \, = \,\Phi \left( {Q_{S - T - B,h} } \right),$$

where $\Phi \left( \cdot \right)$ is the cumulative function of the standard normal distribution. According to Chen et al.^19^, under the assumption of normality and independence, the yield of the entire component product $p_{T}$ is

4 $$p_{T} = \prod\limits_{h = 1}^{s} {p_{h} } = \prod\limits_{h = 1}^{s} {\Phi \left( {Q_{S - T - B,h} } \right)} .$$

Based on Eq. (4), this paper proposed a six sigma product index for the entire component product as follows:

5 $$Q_{{_{S - T - B} }}^{T} = \Phi^{ - 1} \left( {\prod\limits_{h = 1}^{s} {\Phi \left( {Q_{S - T - B,h} } \right)} } \right).$$

According to Eqs. (4) and (5), ${p_{T}} = \Phi \left( C \right)$ when $Q_{{_{S - T - B} }}^{T} = C$. Obviously, there is a one-to-one mathematical relationship between six sigma product index $Q_{{_{S - T - B} }}^{T} = C$ and product yield $p_{T}$. For instance, the number of non-conformities is 1350 ppm, corresponding to the value of the six sigma product index at 3.0.

### Confidence intervals

As noted by Chen et al.^19^, many studies have suggested that companies use control charts to perform process control^20–22^. If the process is under statistical process control, then the quality will be evaluated^23–25^. It is assumed that each subsample contains *n* observations on each quality characteristic, and there are *m* subsamples available. Consequently, for the *ith* subsample of quality characteristic *h*, the sample mean and sample variance are expressed respectively as follows:

6 $$\overline{Y}_{h,i} = \frac{1}{n}\sum\limits_{j = 1}^{n} {Y_{h,i,j} }$$

and

7 $$S_{h,i}^{2} = \frac{1}{n}\sum\limits_{j = 1}^{n} {\left( {Y_{h,i,j} - \overline{Y}_{h,i} } \right)^{2} } ,$$

where $N = m \times n$ denotes the total number of observations. Thus, the estimates of $\delta_{h}$, $\gamma_{h}$ and $Q_{S - T - B,h}$ can be obtained as follows:

8 $$\hat{\delta }_{h} \, = \,\frac{1}{m}\sum\limits_{i = 1}^{m} {\overline{Y}_{h,i} } ,$$

9 $$\hat{\gamma }_{h} = \sqrt {\frac{1}{N - m}\sum\limits_{i = 1}^{m} {nS_{h,i}^{2} } } ,$$

and

10 $$\hat{Q}_{S - T - B,h} \, = \,\frac{{{1} - \hat{\delta }_{h} }}{{\hat{\gamma }_{h} }}.$$

Furthermore, let $Z = \sqrt N {{\left( {\hat{\delta }_{h} - \delta_{h} } \right)} \mathord{\left/ {\vphantom {{\left( {\hat{\delta }_{h} - \delta_{h} } \right)} {\gamma_{h} }}} \right. \kern-0pt} {\gamma_{h} }}$ and $K = {{\left( {N - m} \right)\hat{\gamma }_{h}^{2} } \mathord{\left/ {\vphantom {{\left( {N - m} \right)\hat{\gamma }_{h}^{2} } {\gamma_{h}^{2} }}} \right. \kern-0pt} {\gamma_{h}^{2} }}$. Under the assumption of normality, *Z* and *K* are distributed as $N\left( {0,1} \right)$ and $\chi_{N - m}^{2}$ respectively. Therefore,

$P\left\{ { - Z_{{{{\alpha^{\prime}} \mathord{\left/ {\vphantom {{\alpha^{\prime}} 2}} \right. \kern-0pt} 2}}} \le Z \le Z_{{{{\alpha^{\prime}} \mathord{\left/ {\vphantom {{\alpha^{\prime}} 2}} \right. \kern-0pt} 2}}} } \right\} = \sqrt {1 - \alpha }$ and $P\left\{ {\chi_{{{{\alpha^{\prime}} \mathord{\left/ {\vphantom {{\alpha^{\prime}} 2}} \right. \kern-0pt} 2};N - m}}^{2} \le K \le \chi_{{1 - \left( {{{\alpha^{\prime}} \mathord{\left/ {\vphantom {{\alpha^{\prime}} 2}} \right. \kern-0pt} 2}} \right);N - m}}^{2} } \right\} = \sqrt {1 - \alpha }$, where $Z_{{{{\alpha^{\prime}} \mathord{\left/ {\vphantom {{\alpha^{\prime}} 2}} \right. \kern-0pt} 2}}}$ is the upper ${{\alpha^{\prime}} \mathord{\left/ {\vphantom {{\alpha^{\prime}} 2}} \right. \kern-0pt} 2}$ quantile of $N\left( {0,1} \right)$, $\chi_{{{{\alpha^{\prime}} \mathord{\left/ {\vphantom {{\alpha^{\prime}} 2}} \right. \kern-0pt} 2};N - m}}^{2}$ is the upper ${{\alpha^{\prime}} \mathord{\left/ {\vphantom {{\alpha^{\prime}} 2}} \right. \kern-0pt} 2}$ quantile of $\chi_{N - m}^{2}$, $\alpha^{\prime} = 1 - \sqrt {1 - \alpha }$, and $\sqrt {1 - \alpha }$ represents the confidence level. Since $\hat{\delta }_{h}$ and $\hat{\gamma }_{h}^{2}$ are mutually independent, then *Z* and *K* are also mutually independent. From these relationships, we can further receive the following result:

$$P\left\{ { - Z_{{{{\alpha^{\prime}} \mathord{\left/ {\vphantom {{\alpha^{\prime}} 2}} \right. \kern-0pt} 2}}} \le Z \le Z_{{{{\alpha^{\prime}} \mathord{\left/ {\vphantom {{\alpha^{\prime}} 2}} \right. \kern-0pt} 2}}} ,\chi_{{{{\alpha^{\prime}} \mathord{\left/ {\vphantom {{\alpha^{\prime}} 2}} \right. \kern-0pt} 2};N - m}}^{2} \le K \le \chi_{{1 - \left( {{{\alpha^{\prime}} \mathord{\left/ {\vphantom {{\alpha^{\prime}} 2}} \right. \kern-0pt} 2}} \right);N - m}}^{2} } \right\} = 1 - \alpha .$$

Equivalently,

$$P\left\{ {\hat{\delta }_{h} - Z_{{{{\alpha^{\prime}} \mathord{\left/ {\vphantom {{\alpha^{\prime}} 2}} \right. \kern-0pt} 2}}} \times \left( {\frac{{\gamma_{h} }}{\sqrt N }} \right) \le \delta_{h} \le \hat{\delta }_{h} + Z_{{{{\alpha^{\prime}} \mathord{\left/ {\vphantom {{\alpha^{\prime}} 2}} \right. \kern-0pt} 2}}} \times \left( {\frac{{\gamma_{h} }}{\sqrt N }} \right),\sqrt {\frac{N - m}{{\chi_{{1 - \left( {{{\alpha^{\prime}} \mathord{\left/ {\vphantom {{\alpha^{\prime}} 2}} \right. \kern-0pt} 2}} \right);N - m}}^{2} }}} \hat{\gamma }_{h} \le \gamma_{h} \le \sqrt {\frac{N - m}{{\chi_{{{{\alpha^{\prime}} \mathord{\left/ {\vphantom {{\alpha^{\prime}} 2}} \right. \kern-0pt} 2};N - m}}^{2} }}} \hat{\gamma }_{h} } \right\} = 1 - \alpha$$

Therefore, the confidence region can be denoted as:

11 $$CR\left( {\delta_{h} ,\gamma_{h} } \right) = \left\{ {\left. {\left( {\delta_{h} ,\gamma_{h} } \right)} \right|\hat{\delta }_{h} - e_{h} \le \delta_{h} \le \hat{\delta }_{h} + e_{h} ,\gamma_{Lh} \le \gamma_{h} \le \gamma_{Uh} } \right\},$$

where

12 $$e_{h} = Z_{{0.5 - \sqrt {1 - \alpha } /2}} \times \left( {\frac{{\gamma_{h} }}{\sqrt N }} \right),$$

13 $$\gamma_{Lh} = \sqrt {\frac{N - m}{{\chi_{{0.5 + \sqrt {1 - \alpha } /2;N - m}}^{2} }}} \hat{\gamma }_{h} ,$$

and

14 $$\gamma_{Uh} = \sqrt {\frac{N - m}{{\chi_{{0.5 - \sqrt {1 - \alpha } /2;N - m}}^{2} }}} \hat{\gamma }_{h} .$$

Obviously, index $$Q_{S - T - B,h}$$ is the function of $$\left( {\delta_{h} ,\gamma_{h} } \right)$$. This study adopted the six sigma quality index $$Q_{S - T - B,h} \left( {\delta_{h} ,\gamma_{h} } \right)$$ as the object function and the confidence region $$CR\left( {\delta_{h} ,\gamma_{h} } \right)$$ as the feasible solution area. For any $$\gamma_{h} \le \gamma_{Uh}$$, then $$Q_{S - T - B,h} \left( {\delta_{h} ,\gamma_{h} } \right)$$$$\ge$$$$Q_{S - T - B,h} \left( {\delta_{h} ,\gamma_{Uh} } \right)$$. Thus, the mathematical programming (MP) model for the lower confidence limit is demonstrated below:

15 $$\left\{ \begin{gathered} LQ_{S - T - B,h} = Min \, {{\left( {{1} - \delta_{h} } \right)} \mathord{\left/ {\vphantom {{\left( {{1} - \delta_{h} } \right)} {\gamma_{h} }}} \right. \kern-0pt} {\gamma_{h} }} \hfill \\ subject \, to \hfill \\ \delta_{h} - e_{Uh} \le \delta_{h} \le \hat{\delta }_{h} + e_{Uh} \hfill \\ \end{gathered} \right.,$$

where $LQ_{S - T - B,h}$ is the lower confidence limit of index $Q_{S - T - B,h}$ and

16 $$e_{Uh} = \frac{{Z_{{0.5 - \sqrt {1 - \alpha } /2}} }}{{\sqrt {\chi_{{0.5 - \sqrt {1 - \alpha } /2;N - m}}^{2} } }}\hat{\gamma }_{h} .$$

For any $$\delta_{h} \le \hat{\delta }_{h} + e_{Uh}$$, then $$Q_{S - T - B,h} \left( {\delta_{h} ,\gamma_{h} } \right)$$$$\ge$$$$Q_{S - T - B,h} \left( {\hat{\delta }_{h} + e_{Uh} ,\gamma_{Uh} } \right)$$. Thus, the $$100\left( {1 - \alpha } \right)\%$$ lower confidence limit of $$Q_{S - T - B,h}$$ is presented as follows:

17 $$LQ_{S - T - B,h} \, = \,\frac{{{1} - \left( {\hat{\delta }_{h} + e_{Uh} } \right)}}{{\gamma_{Uh} }}\, = \,\hat{Q}_{S - T - B,h} \sqrt {\frac{{\chi_{{0.5 - \sqrt {1 - \alpha } /2;N - m}}^{2} }}{N - m}} - \frac{{Z_{{0.5 - \sqrt {1 - \alpha } /2}} }}{{\sqrt {N - m} }}.$$

Similarly, for any $$\gamma_{h} \ge \gamma_{Uh}$$, then $$Q_{S - T - B,h} \left( {\delta_{h} ,\gamma_{h} } \right)$$$$\le$$$$Q_{S - T - B,h} \left( {\delta_{h} ,\gamma_{Lh} } \right)$$, and the MP model for the upper confidence limit is exhibited below:

18 $$\left\{ \begin{gathered} UQ_{S - T - B,h} = Max \, {{\left( {{1} - \delta_{h} } \right)} \mathord{\left/ {\vphantom {{\left( {{1} - \delta_{h} } \right)} {\gamma_{h} }}} \right. \kern-0pt} {\gamma_{h} }} \hfill \\ subject \, to \hfill \\ \delta_{h} - e_{Lh} \le \delta_{h} \le \hat{\delta }_{h} + e_{Lh} \hfill \\ \end{gathered} \right.,$$

where $UQ_{S - T - B,h}$ is the upper confidence limit of index $Q_{S - T - B,h}$ and

19 $$e_{Lh} = \frac{{Z_{{0.5 - \sqrt {1 - \alpha } /2}} }}{{\sqrt {\chi_{{0.5 + \sqrt {1 - \alpha } /2;n - 1}}^{2} } }}\hat{\gamma }_{h} .$$

For any $$\delta_{h} \ge \hat{\delta }_{h} - e_{Lh}$$, then $$Q_{S - T - B,h} \left( {\delta_{h} ,\gamma_{h} } \right)$$$$\le$$$$Q_{S - T - B,h} \left( {\hat{\delta }_{h} - e_{Lh} ,\gamma_{Lh} } \right)$$. Since $$UQ_{S - T - B,h}$$$$= {{\left( {{1} - \left( {\delta_{h} - e_{Lh} } \right)} \right)} \mathord{\left/ {\vphantom {{\left( {{1} - \left( {\delta_{h} - e_{Lh} } \right)} \right)} {\gamma_{Lh} }}} \right. \kern-0pt} {\gamma_{Lh} }}$$, then the $$100\left( {1 - \alpha } \right)\%$$ upper confidence limit of $$Q_{S - T - B,h}$$ is displayed below:

20 $$UQ_{S - T - B,h} \, = \,\hat{Q}_{S - T - B,h} \sqrt {\frac{{\chi_{{0.5 + \sqrt {1 - \alpha } /2;N - m}}^{2} }}{N - m}} + \frac{{Z_{{0.5 - \sqrt {1 - \alpha } /2}} }}{{\sqrt {N - m} }}.$$

According to Eqs. (17) and (20), $\left[ {LQ_{S - T - B,h} ,UQ_{S - T - B,h} } \right]$ is the $100\left( {1 - \alpha } \right)\%$ confidence interval of the six sigma quality index for quality characteristic *h*, where *h* $=$ 1, 2, …, s. Based on the above and Chen et al.^19^, we suppose that $\left[ {LQ_{{_{S - T - B} }}^{T} ,Q_{{_{S - T - B} }}^{T} } \right]$ is the $100\left( {1 - \alpha } \right)\%$ confidence interval of the six sigma product index, where

21 $$LQ_{{_{S - T - B} }}^{T} = \Phi^{ - 1} \left( {\prod\limits_{h = 1}^{s} {\Phi \left( {LQ_{S - T - B,h} } \right)} } \right)$$

and

22 $$UQ_{{_{S - T - B} }}^{T} = \Phi^{ - 1} \left( {\prod\limits_{h = 1}^{s} {\Phi \left( {UQ_{S - T - B,h} } \right)} } \right).$$

Then, this paper tested whether the quality of component products met the requirements of the quality level based on Eq. (21) and simultaneously proposed an improvement testing model based on Eqs. (20) and (21).

## Quality evaluation and improvement testing model of component products

The statistical testing method based on the upper confidence limit of the six sigma product index is an effective approach to determining whether the quality is acceptable or needs to be boosted. Hence, this paper explores (1) quality evaluation of component products and (2) an improvement testing model.

### Quality evaluation of component products

To determine whether the value of the six sigma product index is bigger than or equal to *k*, we have to take into account the following two hypotheses: the null hypothesis versus the alternative hypothesis, that is, $H_{0} :Q_{S - T - B}^{T} = k$ versus $H_{1} :Q_{S - T - B}^{T} \ne k$. Then, the statistical testing rules are made as follows:

1. If $LQ_{{_{S - T - B} }}^{T} \le k \le UQ_{{_{S - T - B} }}^{T}$, then do not reject $H_{0}$ and conclude that $Q_{S - T - B}^{T} = k$. That shows the quality of component products must be maintained.
2. If $LQ_{{_{S - T - B} }}^{T} > k$, then reject $H_{0}$ and conclude that $Q_{S - T - B}^{T} > k$. That indicates the quality of component products is greater than the required quality level, so that the quality must be maintained or examined to see if the process tolerance is too loose.
3. If $UQ_{{_{S - T - B} }}^{T}$ $<$ k, then reject $H_{0}$ and conclude that $Q_{S - T - B}^{T} < k$. That means the quality of component products must be boosted.
  23 $$LQ_{{_{S - T - B} }}^{T} = \Phi^{ - 1} \left( {\prod\limits_{h = 1}^{s} {\Phi \left( {\hat{Q}_{S - T - B,h} \sqrt {\frac{{\chi_{{0.5 - \sqrt {1 - \alpha } /2;N - m}}^{2} }}{N - m}} - \frac{{Z_{{0.5 - \sqrt {1 - \alpha } /2}} }}{{\sqrt {N - m} }}} \right)} } \right)$$
  and
  24 $$UQ_{{_{S - T - B} }}^{T} = \Phi^{ - 1} \left( {\prod\limits_{h = 1}^{s} {\Phi \left( {\hat{Q}_{S - T - B,h} \sqrt {\frac{{\chi_{{0.5 + \sqrt {1 - \alpha } /2;N - m}}^{2} }}{N - m}} + \frac{{Z_{{0.5 - \sqrt {1 - \alpha } /2}} }}{{\sqrt {N - m} }}} \right)} } \right).$$

For practical and easier application, the following steps are taken to illustrate the application procedures for the lower and upper confidence limits of the six sigma product index presented above.

Step 1: Calculate the values of $\hat{\delta }_{h}$, $\hat{\gamma }_{h}$ and $\hat{Q}_{S - T - B,h}$ based on the overall sample mean and the pooled sample variance for each quality characteristic as follows:

25 $$\hat{\delta }_{h} \, = \,\frac{1}{m}\sum\limits_{i = 1}^{m} {\overline{Y}_{h,i} } ,$$

26 $$\hat{\gamma }_{h} = \sqrt {\frac{1}{N - m}\sum\limits_{i = 1}^{m} {nS_{h,i}^{2} } } ,$$

and

27 $$\hat{Q}_{S - T - B,h} \, = \,\frac{{{1} - \hat{\delta }_{h} }}{{\hat{\gamma }_{h} }}.$$

Step 2: Calculate the values of $\left[ {LQ_{S - T - B,h} ,UQ_{S - T - B,h} } \right]$ for each quality characteristic based on Eqs. (17–20) and Step 1 as follows:

28 $$LQ_{S - T - B,h} \, = \,\hat{Q}_{S - T - B,h} \sqrt {\frac{{\chi_{{0.5 - \sqrt {1 - \alpha } /2;N - m}}^{2} }}{N - m}} - \frac{{Z_{{0.5 - \sqrt {1 - \alpha } /2}} }}{{\sqrt {N - m} }}.$$

and

29 $$UQ_{S - T - B,h} \, = \,\hat{Q}_{S - T - B,h} \sqrt {\frac{{\chi_{{0.5 + \sqrt {1 - \alpha } /2;N - m}}^{2} }}{N - m}} + \frac{{Z_{{0.5 - \sqrt {1 - \alpha } /2}} }}{{\sqrt {N - m} }}.$$

Step 3: According to Eqs. (23), (24) and Step 2, calculate the values of $\left[ {LQ_{{_{S - T - B} }}^{T} ,Q_{{_{S - T - B} }}^{T} } \right]$ as follows:

30 $$LQ_{{_{S - T - B} }}^{T} = \Phi^{ - 1} \left( {\prod\limits_{h = 1}^{s} {\Phi \left( {\hat{Q}_{S - T - B,h} \sqrt {\frac{{\chi_{{0.5 - \sqrt {1 - \alpha } /2;N - m}}^{2} }}{N - m}} - \frac{{Z_{{0.5 - \sqrt {1 - \alpha } /2}} }}{{\sqrt {N - m} }}} \right)} } \right)$$

and

31 $$UQ_{{_{S - T - B} }}^{T} = \Phi^{ - 1} \left( {\prod\limits_{h = 1}^{s} {\Phi \left( {\hat{Q}_{S - T - B,h} \sqrt {\frac{{\chi_{{0.5 + \sqrt {1 - \alpha } /2;N - m}}^{2} }}{N - m}} + \frac{{Z_{{0.5 - \sqrt {1 - \alpha } /2}} }}{{\sqrt {N - m} }}} \right)} } \right).$$

Step 4: According to the statistical testing rules and Step 3, determine whether the quality is acceptable or needs to be leveled up.

### An improvement testing model

When the testing result reveals that the quality does not meet the requirements of the quality level, then the process must be improved. Next, this paper applies the confidence intervals of product indicators before and after improvement and proposes an improvement testing model. It is assumed that the product index value before improvement is $Q_{{_{S - T - B} }}^{T}$ and the product index value after improvement is $Q_{{_{S - T - B} }}^{\prime T}$. To judge whether the improvement is effective, the statistical hypothesis test is expressed as follows:

$$H_{0} :Q_{S - T - B}^{T} = Q_{S - T - B}^{\prime T}$$

versus

$$H_{1} :Q_{S - T - B}^{T} \ne Q_{S - T - B}^{\prime T} .$$

Then, the statistical testing rules are described as follows:

1. If $\left[ {LQ_{{_{S - T - B} }}^{T} ,UQ_{{_{S - T - B} }}^{T} } \right] \cap \left[ {LQ_{S - T - B}^{\prime T} ,UQ_{S - T - B}^{\prime T} } \right] \ne \phi$, then do not reject $H_{0}$ and conclude $Q_{S - T - B}^{T} = Q_{S - T - B}^{\prime T}$. That means the improvement has no significant effect and must continue.
2. If $UQ_{S - T - B}^{\prime T} < LQ_{{_{S - T - B} }}^{T}$, then reject $H_{0}$ and conclude $Q_{S - T - B}^{T} > Q_{S - T - B}^{\prime T}$. That indicates the improvement is not effective, but the quality is even worse, so it is necessary to review and continue to improve.
3. If $UQ_{{_{S - T - B} }}^{T} < LQ_{S - T - B}^{\prime T}$, then reject $H_{0}$ and conclude $Q_{S - T - B}^{T} < Q_{S - T - B}^{\prime T}$. That shows the improvement has achieved remarkable outcomes.

The confidence intervals of $\left[ {LQ_{{_{S - T - B} }}^{T} ,UQ_{{_{S - T - B} }}^{T} } \right]$ for component product indicators before improvement are shown in Eqs. (23) and (24). The confidence intervals of $\left[ {LQ_{S - T - B}^{\prime T} ,UQ_{S - T - B}^{\prime T} } \right]$ for component product indicators after improvement can be received according to Eqs. (23) and (24) using the improved relevant sample data. The detailed calculation process and evaluation steps are depicted as follows:

Step $1^{\prime}$: Calculate the values of $\hat{\delta }^{\prime}_{h}$, $\hat{\gamma }^{\prime}_{h}$ and $\hat{Q}_{S - T - B,h}^{\prime }$ based on the improved population sample mean and the pooled sample variance for each quality characteristic as follows:

32 $$\hat{\delta }^{\prime}_{h} = \frac{1}{m}\sum\limits_{i = 1}^{m} {\overline{Y}^{\prime}_{h,i} } ,$$

33 $$\hat{\gamma }_{h}^{\prime } = \sqrt {\frac{1}{N - m}\sum\limits_{i = 1}^{m} {nS_{h,i}^{\prime 2} } } ,$$

and

34 $$\hat{Q}_{S - T - B,h}^{\prime } \, = \,\frac{{{1} - \hat{\delta }_{h}^{\prime } }}{{\hat{\gamma }_{h}^{\prime } }}.$$

Step $2^{\prime}$: Calculate the values of the improved confidence interval of the six sigma quality index for each quality characteristic, as displayed below:

35 $$LQ_{S - T - B,h}^{\prime } \, = \,\hat{Q}_{S - T - B,h}^{\prime } \sqrt {\frac{{\chi_{{0.5 - \sqrt {1 - \alpha } /2;N - m}}^{2} }}{N - m}} - \frac{{Z_{{0.5 - \sqrt {1 - \alpha } /2}} }}{{\sqrt {N - m} }}$$

and

36 $$UQ_{S - T - B,h}^{\prime } \, = \,\hat{Q}_{S - T - B,h}^{\prime } \sqrt {\frac{{\chi_{{0.5 + \sqrt {1 - \alpha } /2;N - m}}^{2} }}{N - m}} + \frac{{Z_{{0.5 - \sqrt {1 - \alpha } /2}} }}{{\sqrt {N - m} }}.$$

Step $3^{\prime}$: Calculate the values of the improved confidence interval of the six sigma product index, as shown below:

37 $$LQ_{{_{S - T - B} }}^{\prime T} \, = \,\Phi^{ - 1} \left( {\prod\limits_{h = 1}^{s} {\Phi \left( {\hat{Q}_{S - T - B,h}^{\prime } \sqrt {\frac{{\chi_{{0.5 - \sqrt {1 - \alpha } /2;N - m}}^{2} }}{N - m}} - \frac{{Z_{{0.5 - \sqrt {1 - \alpha } /2}} }}{{\sqrt {N - m} }}} \right)} } \right)$$

and

38 $$UQ_{S - T - B}^{\prime T} = \Phi^{ - 1} \left( {\prod\limits_{h = 1}^{s} {\Phi \left( {\hat{Q}_{S - T - B,h}^{\prime } \sqrt {\frac{{\chi_{{0.5 + \sqrt {1 - \alpha } /2;N - m}}^{2} }}{N - m}} + \frac{{Z_{{0.5 - \sqrt {1 - \alpha } /2}} }}{{\sqrt {N - m} }}} \right)} } \right).$$

Step $4^{\prime}$: According to the above-mentioned improved confidence intervals of the six sigma product index as well as the improved and verified statistical testing rules, it is determined whether the manufacturing process of component products has reached the required level, or whether it is necessary to carry out improvements or tolerance reviews.

## An illustrative example

We take the shaft of a fan motor as an example to illustrate the application of the evaluation model proposed in this paper. The shaft of a fan motor has five important quality characteristics (QC), including two types of roundness, concentricity, surface roughness, and perpendicularity. To determine whether the six sigma product index value is bigger than or equal to 5 (*k* $=$ 5), then the null hypothesis is set as $H_{0} :Q_{S - T - B}^{T} = 5$, and the alternative hypothesis is set as $H_{1} :Q_{S - T - B}^{T} \ne 5$. In the case of a significant level at $\alpha =$ 0.01, this paper takes four steps of the statistical testing demonstrated in Sect. “An illustrative example” as follows:

Step 1: Calculate the values of $\hat{\delta }_{h}$, $\hat{\gamma }_{h}$ and $\hat{Q}_{S - T - B,h}$ with $m = 25$ and $n = 11$, *h* $=$ 1, 2, 3, 4, 5 based on the overall sample mean and the pooled sample variance for five important quality characteristics as follows:

QC 1: $$\hat{\delta }_{1} =$$ 0.512, $$\hat{\gamma }_{1} =$$ 0.112, and $$\hat{Q}_{S - T - B,1} =$$ 4.357.

QC 2: $$\hat{\delta }_{2} =$$ 0.511, $$\hat{\gamma }_{2} =$$ 0.113, and $$\hat{Q}_{S - T - B,2} =$$ 4.327.

QC 3: $$\hat{\delta }_{3} =$$ 0.523, $$\hat{\gamma }_{3} =$$ 0.111, and $$\hat{Q}_{S - T - B,3} =$$ 4.297.

QC 4: $$\hat{\delta }_{4} =$$ 0.545, $$\hat{\gamma }_{4} =$$ 0.101, and $$\hat{Q}_{S - T - B,4} =$$ 4.505.

QC 5: $$\hat{\delta }_{5} =$$ 0.523, $$\hat{\gamma }_{5} =$$ 0.114, and $$\hat{Q}_{S - T - B,5} =$$ 4.184.

Step 2: Calculate the values of $\left[ {LQ_{S - T - B,h} ,UQ_{S - T - B,h} } \right]$ based on Step 1 for five important quality characteristics as follows:

### QC 1:

$$LQ_{S - T - B,1} \, = \,\hat{Q}_{S - T - B,1} \sqrt {\frac{{\chi_{0.0025;N - m}^{2} }}{270}} - \frac{{Z_{0.0025} }}{{\sqrt {270} }} = \hat{Q}_{S - T - B,1} \sqrt {\frac{209.341}{{270}}} - \frac{2.807}{{\sqrt {270} }} =$$

4.357 $$\times 0.881 - 0.171 =$$ 3.668

$$UQ_{S - T - B,1} \, = \,\hat{Q}_{S - T - B,1} \sqrt {\frac{{\chi_{0.9975;N - m}^{2} }}{270}} + \frac{{Z_{0.0025} }}{{\sqrt {270} }} = \hat{Q}_{S - T - B,1} \sqrt {\frac{339.824}{{270}}} + \frac{2.807}{{\sqrt {270} }} =$$

4.357 $$\times 1.122 + 0.171 =$$ 5.060

### QC 2:

$$LQ_{S - T - B,2} \, = \,\hat{Q}_{S - T - B,2} \sqrt {\frac{{\chi_{0.0025;N - m}^{2} }}{270}} - \frac{{Z_{0.0025} }}{{\sqrt {270} }} = \,\hat{Q}_{S - T - B,2} \sqrt {\frac{209.341}{{270}}} - \frac{2.807}{{\sqrt {270} }} =$$

4.327 $$\times 0.881 - 0.171 =$$ 3.641

$$UQ_{S - T - B,2} \, = \,\hat{Q}_{S - T - B,2} \sqrt {\frac{{\chi_{0.9975;N - m}^{2} }}{270}} + \frac{{Z_{0.0025} }}{{\sqrt {270} }} = \hat{Q}_{S - T - B,2} \sqrt {\frac{339.824}{{270}}} + \frac{2.807}{{\sqrt {270} }} =$$

4.327 $$\times 1.122 + 0.171 =$$ 5.026

### QC 3:

$$LQ_{S - T - B,3} \, = \,\hat{Q}_{S - T - B,3} \sqrt {\frac{{\chi_{0.0025;N - m}^{2} }}{270}} - \frac{{Z_{0.0025} }}{{\sqrt {270} }} = \hat{Q}_{S - T - B,3} \sqrt {\frac{209.341}{{270}}} - \frac{2.807}{{\sqrt {270} }} =$$

4.297 $$\times 0.881 - 0.171 =$$ 3.615

$$UQ_{S - T - B,3} \, = \,\hat{Q}_{S - T - B,3} \sqrt {\frac{{\chi_{0.9975;N - m}^{2} }}{270}} + \frac{{Z_{0.0025} }}{{\sqrt {270} }} = \hat{Q}_{S - T - B,3} \sqrt {\frac{339.824}{{270}}} + \frac{2.807}{{\sqrt {270} }} =$$

4.297 $$\times 1.122 + 0.171 =$$ 4.992

### QC 4:

$$LQ_{S - T - B,4} \, = \,\hat{Q}_{S - T - B,4} \sqrt {\frac{{\chi_{0.0025;N - m}^{2} }}{270}} - \frac{{Z_{0.0025} }}{{\sqrt {270} }} = \,\hat{Q}_{S - T - B,4} \sqrt {\frac{209.341}{{270}}} - \frac{2.807}{{\sqrt {270} }} =$$

4.505 $$\times 0.881 - 0.171 =$$ 4.140

$$UQ_{S - T - B,4} \, = \,\hat{Q}_{S - T - B,4} \sqrt {\frac{{\chi_{0.9975;N - m}^{2} }}{270}} + \frac{{Z_{0.0025} }}{{\sqrt {270} }} = \,\hat{Q}_{S - T - B,4} \sqrt {\frac{339.824}{{270}}} + \frac{2.807}{{\sqrt {270} }} =$$

4.505 $$\times 1.122 + 0.171 =$$ 5.225

### QC 5:

$$LQ_{S - T - B,5} \, = \,\hat{Q}_{S - T - B,5} \sqrt {\frac{{\chi_{0.0025;N - m}^{2} }}{270}} - \frac{{Z_{0.0025} }}{{\sqrt {270} }} = \hat{Q}_{S - T - B,5} \sqrt {\frac{209.341}{{270}}} - \frac{2.807}{{\sqrt {270} }} =$$

3.92 $$\times 0.881 - 0.171 =$$ 3.283

$$UQ_{S - T - B,5} \, = \,\hat{Q}_{S - T - B,5} \sqrt {\frac{{\chi_{0.9975;N - m}^{2} }}{270}} + \frac{{Z_{0.0025} }}{{\sqrt {270} }} = \hat{Q}_{S - T - B,5} \sqrt {\frac{339.824}{{270}}} + \frac{2.807}{{\sqrt {270} }} =$$

3.92 $$\times 1.122 + 0.171 =$$ 4.570

Step 3: Calculate the values of $\left[ {LQ_{{_{S - T - B} }}^{T} ,Q_{{_{S - T - B} }}^{T} } \right]$ based on the 99% confidence intervals of five important quality characteristics, as depicted below:

$$LQ_{{_{S - T - B} }}^{T} = \Phi^{ - 1} \left( {\prod\limits_{h = 1}^{5} {\Phi \left( {\hat{Q}_{S - T - B,h} \sqrt {\frac{209.341}{{270}}} - \frac{2.807}{{\sqrt {270} }}} \right)} } \right)$$ $$=$$ 3.109.and

$$UQ_{{_{S - T - B} }}^{T} = \Phi^{ - 1} \left( {\prod\limits_{h = 1}^{5} {\Phi \left( {\hat{Q}_{S - T - B,h} \sqrt {\frac{339.824}{{270}}} + \frac{2.807}{{\sqrt {270} }}} \right)} } \right)$$ $$=$$ 4.507.

Step 4: According to the statistical testing rules and Step 3, it is determined whether the quality is acceptable or needs to improve. Also, based on the statistical testing rule (3), because $UQ_{{_{S - T - B} }}^{T}$ $<$ k, then reject $H_{0}$ and conclude $Q_{S - T - B}^{T} < k$, showing that the quality of component products must be improved.

Obviously, according to the above-stated evaluation result, the shaft of the fan motor does not meet the required level quality, so the process must be improved. As mentioned above, in order to verify the effectiveness of improvement, this paper performs the following steps according to the steps of improvement and testing stated in Sect. “An illustrative example”:

Step $1^{\prime}$: Calculate the values of $\hat{\delta }^{\prime}_{h}$, $\hat{\gamma }^{\prime}_{h}$ and $\hat{Q}^{\prime}_{S - T - B,h}$ with $m = 25$ and $n = 11$, *h* $=$ 1, 2, 3, 4, 5 based on the improved population sample mean and the pooled sample variance of five quality characteristics as follows:

QC 1: $$\hat{\delta }^{\prime}_{1} =$$ 0.512, $$\hat{\gamma }^{\prime}_{1} =$$ 0.081, and $$\hat{Q}^{\prime}_{{S - T - B,{1}}} =$$ 6.025.

QC 2: $$\hat{\delta }^{\prime}_{2} =$$ 0.511, $$\hat{\gamma }^{\prime}_{2} =$$ 0.072, and $$\hat{Q}^{\prime}_{{S - T - B,{2}}} =$$ 6.857.

QC 3: $$\hat{\delta }^{\prime}_{3} =$$ 0.510, $$\hat{\gamma }^{\prime}_{3} =$$ 0.080, and $$\hat{Q}^{\prime}_{S - T - B,3} =$$ 6.125.

QC 4: $$\hat{\delta }^{\prime}_{4} =$$ 0.513, $$\hat{\gamma }^{\prime}_{4} =$$ 0.078, and $$\hat{Q}^{\prime}_{S - T - B,4} =$$ 6.244.

QC 5: $$\hat{\delta }^{\prime}_{5} =$$ 0.514, $$\hat{\gamma }^{\prime}_{5} =$$ 0.079, and $$\hat{Q}^{\prime}_{S - T - B,5} =$$ 6.152.

Step $2^{\prime}$: Based on Step $1^{\prime}$, calculate the values of the improved confidence interval of the six sigma quality index for each quality characteristic, as depicted below:

### QC 1:

$$LQ_{S - T - B,1}^{\prime } \, = \,\hat{Q}_{{S - T - B,{1}}}^{\prime } \sqrt {\frac{{\chi_{0.0025;N - m}^{2} }}{270}} - \frac{{Z_{0.0025} }}{{\sqrt {270} }} = \hat{Q}_{{S - T - B,{1}}}^{\prime } \sqrt {\frac{209.341}{{270}}} - \frac{2.807}{{\sqrt {270} }} =$$

6.025 $$\times 0.881 - 0.171 =$$ 5.137

$$UQ_{S - T - B,1}^{\prime } \, = \,\hat{Q}_{{S - T - B,{1}}}^{\prime } \sqrt {\frac{{\chi_{0.9975;N - m}^{2} }}{270}} + \frac{{Z_{0.0025} }}{{\sqrt {270} }} = \hat{Q}_{{S - T - B,{1}}}^{\prime } \sqrt {\frac{339.824}{{270}}} + \frac{2.807}{{\sqrt {270} }} =$$

6.025 $$\times 1.122 + 0.171 =$$ 6.931

### QC 2:

$$LQ^{\prime}_{S - T - B,2} \, = \,\hat{Q}_{{S - T - B,{2}}}^{\prime } \sqrt {\frac{{\chi_{0.0025;N - m}^{2} }}{270}} - \frac{{Z_{0.0025} }}{{\sqrt {270} }} = \hat{Q}_{{S - T - B,{2}}}^{\prime } \sqrt {\frac{209.341}{{270}}} - \frac{2.807}{{\sqrt {270} }} =$$

6.857 $$\times 0.881 - 0.171 =$$ 5.870

$$UQ_{S - T - B,2}^{\prime } \, = \,\hat{Q}_{{S - T - B,{2}}}^{\prime } \sqrt {\frac{{\chi_{0.9975;N - m}^{2} }}{270}} + \frac{{Z_{0.0025} }}{{\sqrt {270} }} = \hat{Q}_{{S - T - B,{2}}}^{\prime } \sqrt {\frac{339.824}{{270}}} + \frac{2.807}{{\sqrt {270} }} =$$

6.857 $$\times 1.122 + 0.171 =$$ 7.865

### QC 3:

$$LQ_{S - T - B,3}^{\prime } \, = \,\hat{Q}_{{S - T - B,{3}}}^{\prime } \sqrt {\frac{{\chi_{0.0025;N - m}^{2} }}{270}} - \frac{{Z_{0.0025} }}{{\sqrt {270} }} = \hat{Q}_{{S - T - B,{3}}}^{\prime } \sqrt {\frac{209.341}{{270}}} - \frac{2.807}{{\sqrt {270} }} =$$

6.125 $$\times 0.881 - 0.171 =$$ 5.225

$$UQ_{S - T - B,3}^{\prime } \, = \,\hat{Q}_{{S - T - B,{3}}}^{\prime } \sqrt {\frac{{\chi_{0.9975;N - m}^{2} }}{270}} + \frac{{Z_{0.0025} }}{{\sqrt {270} }} = \hat{Q}_{{S - T - B,{3}}}^{\prime } \sqrt {\frac{339.824}{{270}}} + \frac{2.807}{{\sqrt {270} }} =$$

6.125 $$\times 1.122 + 0.171 =$$ 7.043

### QC 4:

$$LQ_{S - T - B,4}^{\prime } \, = \,\hat{Q}_{{S - T - B,{4}}}^{\prime } \sqrt {\frac{{\chi_{0.0025;N - m}^{2} }}{270}} - \frac{{Z_{0.0025} }}{{\sqrt {270} }} = \hat{Q}_{{S - T - B,{4}}}^{\prime } \sqrt {\frac{209.341}{{270}}} - \frac{2.807}{{\sqrt {270} }} =$$

6.244 $$\times 0.881 - 0.171 =$$ 5.330

$$UQ_{S - T - B,4}^{\prime } \, = \,\hat{Q}_{{S - T - B,{4}}}^{\prime } \sqrt {\frac{{\chi_{0.9975;N - m}^{2} }}{270}} + \frac{{Z_{0.0025} }}{{\sqrt {270} }} = \hat{Q}_{{S - T - B,{4}}}^{\prime } \sqrt {\frac{339.824}{{270}}} + \frac{2.807}{{\sqrt {270} }} =$$

6.244 $$\times 1.122 + 0.171 =$$ 7.177

### QC 5:

$$LQ_{S - T - B,5}^{\prime } \, = \,\hat{Q}_{{S - T - B,{5}}}^{\prime } \sqrt {\frac{{\chi_{0.0025;N - m}^{2} }}{270}} - \frac{{Z_{0.0025} }}{{\sqrt {270} }} = \hat{Q}_{{S - T - B,{5}}}^{\prime } \sqrt {\frac{209.341}{{270}}} - \frac{2.807}{{\sqrt {270} }} =$$

6.152 $$\times 0.881 - 0.171 =$$ 5.249

$$UQ_{S - T - B,5}^{\prime } \, = \,\hat{Q}_{{S - T - B,{5}}}^{\prime } \sqrt {\frac{{\chi_{0.9975;N - m}^{2} }}{270}} + \frac{{Z_{0.0025} }}{{\sqrt {270} }} = \hat{Q}_{{S - T - B,{5}}}^{\prime } \sqrt {\frac{339.824}{{270}}} + \frac{2.807}{{\sqrt {270} }} =$$

6.152 $$\times 1.122 + 0.171 =$$ 7.074

Step $3^{\prime}$: Calculate the values of $\left[ {LQ_{S - T - B}^{\prime T} ,Q_{S - T - B}^{\prime T} } \right]$ based on the 99% confidence intervals of five important quality characteristics below:

$$LQ_{S - T - B}^{\prime T} = \Phi^{ - 1} \left( {\prod\limits_{h = 1}^{5} {\Phi \left( {\hat{Q}^{\prime}_{S - T - B,h} \sqrt {\frac{209.341}{{270}}} - \frac{2.807}{{\sqrt {270} }}} \right)} } \right)$$ $$=$$ 4.959. and

$$UQ_{S - T - B}^{\prime T} = \Phi^{ - 1} \left( {\prod\limits_{h = 1}^{5} {\Phi \left( {\hat{Q}_{S - T - B,h}^{\prime } \sqrt {\frac{339.824}{{270}}} + \frac{2.807}{{\sqrt {270} }}} \right)} } \right)$$ $$=$$ 6.834.

Step $4^{\prime}$: Based on the statistical testing rule (3), since $UQ_{{_{S - T - B} }}^{T} < LQ_{S - T - B}^{\prime T}$, then reject $H_{0}$ and conclude $Q_{S - T - B}^{T} < Q_{S - T - B}^{\prime T}$, showing that significant improvements have been made.

## Conclusions and future scope of research

Many important parts of tool machines have key STB quality characteristics. This paper proposed a complete testing model of evaluation and improvement for a number of component products containing the STB-type quality characteristics, contributing to raising the product quality of machine tools as well as boosting the quality of component products processed by machine tools. This model can not only increase product value in the industry chain but also lower environmental pollution caused by rework or scrap, beneficial for enterprises to better their image of taking full responsibility for society. To avoid losing generality, in this paper, we assumed that the component products processed by the machine tools had STB-type quality characteristics. Additionally, we also integrated and evaluated the STB-type six sigma quality indexes for all STB-type quality characteristics and then proposed a six sigma product index which had a one-to-one mathematical relationship with the product yield index. First, this paper derived the 100($1 - \alpha$)% confidence intervals of the six sigma quality index for each quality characteristic based on in-control data. Next, based on all confidence intervals of the six sigma quality index, we derived the 100($1 - \alpha$)% confidence intervals of the six sigma product index. We also used the confidence intervals of the six sigma product index to evaluate whether the component products meet the required quality level. When the evaluation result did not meet the requirements of the quality level, then the process must be improved. Subsequently, the confidence intervals of the six-sigma product index before and after improvement were employed to test the quality levels before and after improvement to see if there was any significant difference, so as to verify the effectiveness of the improvement.

Clearly, the model in this paper was built on the assumption that the process was normal and the quality characteristics were mutually independent. According to a number of studies, some process distributions are non-normal, and the quality characteristics are not necessarily mutually independent. Hence, the non-normal processes and relationships between quality characteristics that are not mutually independent can be further discussed in the future research scope and focus so as to make the entire model more complete.

## Data Availability

All data generated or analyzed during this study are included in the article.
